# Life course longitudinal growth and risk of knee osteoarthritis at age 53 years: evidence from the 1946 British birth cohort study

**DOI:** 10.1016/j.joca.2020.12.012

**Published:** 2021-03

**Authors:** K.A. Staines, R. Hardy, H.J. Samvelyan, K.A. Ward, R. Cooper

**Affiliations:** † School of Pharmacy and Biomolecular Sciences, University of Brighton, Brighton, UK; ‡ School of Applied Sciences, Edinburgh Napier University, Edinburgh UK; § Cohort and Longitudinal Studies Enhancement Resources (CLOSER), UCL Institute of Education, London, UK; ‖ MRC Lifecourse Epidemiology, Human Development and Health, University of Southampton, Southampton, UK; ¶ Department of Sport and Exercise Sciences, Musculoskeletal Science and Sports Medicine Research Centre, Manchester Metropolitan University, Manchester, UK

**Keywords:** Osteoarthritis, SITAR, Growth, Life course, Birth cohort

## Abstract

**Objective:**

To examine the relationship between height gain across childhood and adolescence with knee osteoarthritis in the MRC National Survey of Health and Development (NSHD).

**Materials and methods:**

Data are from 3035 male and female participants of the NSHD. Height was measured at ages 2, 4, 6, 7, 11 and 15 years, and self-reported at ages 20 years. Associations between (1) height at each age (2) height gain during specific life periods (3) Super-Imposition by Translation And Rotation (SITAR) growth curve variables of height size, tempo and velocity, and knee osteoarthritis at 53 years were tested.

**Results:**

In sex-adjusted models, estimated associations between taller height and decreased odds of knee osteoarthritis at age 53 years were small at all ages - the largest associations were an OR of knee osteoarthritis of 0.9 per 5 cm increase in height at age 4, (95% CI 0.7–1.1) and an OR of 0.9 per 5 cm increase in height, (95% CI 0.8–1.0) at age 6. No associations were found between height gain during specific life periods or the SITAR growth curve variables and odds of knee osteoarthritis.

**Conclusions:**

There was limited evidence to suggest that taller height in childhood is associated with decreased odds of knee osteoarthritis at age 53 years in this cohort. This work enhances our understanding of osteoarthritis predisposition and the contribution of life course height to this.

## Introduction

Joint health is reliant upon the preservation of the articular cartilage and, its degradation is one of the main hallmarks of the degenerative joint disease osteoarthritis. Osteoarthritis, characterised by articular cartilage loss, subchondral bone thickening and osteophyte formation, is a major health care burden throughout the world. It is estimated that worldwide at least 10% of men and 18% of women aged over 60 years have symptomatic osteoarthritis. Osteoarthritis causes much pain and disability, and yet its underlying molecular mechanisms are not fully understood. Indeed, even the precipitating pathology remains a matter of debate and we are still unable to identify those at most risk of developing the disease.

Our previous work in a spontaneous murine model of ageing-related osteoarthritis, the STR/Ort mouse, revealed accelerated long bone growth, increased growth plate chondrocyte differentiation, and widespread abnormal expression of chondrocyte markers in osteoarthritis-prone mice^1^. Furthermore, we revealed enriched growth plate bridging, indicative of advanced and thus premature growth plate closure, in these mice^1^. Together this suggested that osteoarthritis development is associated with an accelerated growth phenotype and advanced pubertal onset.

Consistent with this finding, canine hip dysplasia (a hereditary predisposition to degenerative osteoarthritis) is more common in certain breeds, in particular larger breeds which tend to grow more rapidly^2^. However, associations between lifetime linear growth, i.e., height gain during specific life periods up to the attainment of adult height, and knee osteoarthritis development in human populations have, to our knowledge, not yet been studied. Previous epidemiological analyses of the Hertfordshire Cohort Study and the Medical Research Council National Survey of Health and Development (MRC NSHD) have found associations between low birth weight and high body mass index across life and increased risk of developing osteoarthritis^3^^,^^4^. This therefore suggests that life course size may predispose to osteoarthritis later in life.

Herein, we use one of these studies, the MRC NSHD, to examine the relationship between childhood and adolescent height growth and knee osteoarthritis at 53 years. Our aims were to: (1) test associations between height at different ages in early life and knee osteoarthritis in adulthood; (2) assess how patterns of height growth during childhood and adolescence are associated with knee osteoarthritis.

## Method

### Study sample

The MRC NSHD is a birth cohort study, which includes a nationally representative sample of 2,815 men and 2,547 women born in England, Scotland, and Wales during 1 week in March 1946. The cohort has been followed prospectively across life with outcome data for these analyses drawn from a data collection in 1999, when participants were 53 years old^5^. At 53, 3035 participants (1,472 men, 1,563 women) participated, the majority (*n* = 2,989) were interviewed and examined in their own homes by research nurses with others completing a postal questionnaire (*n* = 46). The responding sample at age 53 is in most respects representative of the national population of a similar age^6^. The data collection at age 53 years received ethical approval from the North Thames Multi-centre Research Ethics Committee, and written informed consent was given by all respondents.

### Outcome – knee osteoarthritis

During the home visit at age 53 years, trained nurses conducted clinical examinations of study participants’ knees^3^. Based on these examinations, the American College of Rheumatology criteria for the clinical diagnosis of idiopathic knee osteoarthritis were used to identify those with knee pain in either knee on most days for at least 1 month in the last year prior to the examination in 1999, and at least two of the following: stiffness, crepitus, bony tenderness and bony enlargement^7^.

### Height variables

Height was measured by nurses using standardised protocols at ages 2, 4, 7, 11, and 15 years, and self-reported at age 20. Individual patterns of height growth during puberty were estimated using the SuperImposition by Translation and Rotation (SITAR) model of growth curve analysis, as previously described by Cole *et al.*^9^^,^^10^ The SITAR model estimates the mean growth curve and three individual-specific parameters: size (reflecting differences in mean height), tempo (reflecting differences in the timing of the pubertal growth spurt) and velocity (reflecting differences in the duration of the growth spurt), each expressed relative to the mean curve.

### Covariates

Factors that may potentially confound the main associations of interest were selected *a priori* based on previous findings in the literature^3^. These were birth weight, father's occupational class in childhood (categorised as non-manual vs manual) and sporting ability at 13 years (categorised as above average, average, or below average according to teacher reports of their sporting ability)^11^^,^^12^. Weight was measured by nurses using standardised protocols at ages 2, 4, 7, 11, and 15 years, and self-reported at age 20.

### Statistical analysis

To address the two main aims, we used logistic regression models to test associations between: (1) height at each age (aim 1); (2) conditional changes in height during specific life periods (early childhood: 2–4 years; late childhood: 4–7 years; childhood to adolescence: 7–15 years; adolescence to young adulthood: 15–20 years) (aim 2) and; (3) each SITAR height variable (aim 2) and odds ratios (ORs) of knee osteoarthritis. In models to address aim 2, we generated conditional changes in height by regressing each height measure on the earlier height measure for each sex and calculating the residuals^13^. The residuals were standardized (to have mean 0 and SD of 1) to ensure their comparability and these were included as the main independent variables. In initial models, we formally tested for interactions between sex and each main independent variable and where no evidence of interaction was found based on statistical significance (*P* < 0.05), models were fitted with men and women combined and adjusted for sex. We also tested for deviations from linearity by including quadratic terms, but there was no evidence of this. In each set of models we first adjusted for sex (where there was no evidence of interaction), before then also adjusting for early life factors (birth weight + sporting ability at 13 years + father's occupational class in childhood). In our final model, we adjusted for weight at each age for aim 1, conditional weight gain (aim 2) and the SITAR weight variables (aim 2) to assess the contribution of weight during growth. To maximise statistical power, each set of models were run on the sample with valid data for the outcome, the specified independent variable and the covariates for that analysis. Data were analysed using Stata statistical software (version SE 14.2).

### Sensitivity analyses

To assess the potential impact of having to exclude those participants lost to follow-up before age 53 years and with missing data, comparisons were made between those included and those excluded from the main analyses. In addition, the sex-adjusted analyses were rerun in the maximum available samples including all available participants rather than being restricted to the sample with valid data on all measures. To assess the influence of potential secondary osteoarthritis on our findings the main analyses were repeated after excluding those participants with knee osteoarthritis who had reported ever seeing a doctor about an injury to the knee in which osteoarthritis was diagnosed. Finally, sex stratified analyses were run.

## Results

### Cohort characteristics

A total of 1,437 men and 1,478 women had complete data on the SITAR parameters of height and knee osteoarthritis. Descriptive statistics are described in Table I. In this sample, the percentage of individuals with knee osteoarthritis at 53 years of age was higher in women (13.1%) than in men (7.3%).

**Table I:** Characteristics of the sample from the MRC National Survey of Health and Development with complete data on the SITAR height parameters and the outcome, knee osteoarthritis

	Men			Women		
	N	Mean	SD	n	Mean	SD
Height 2 years (cm)	1,211	85.91	5.24	1,197	84.72	4.57
Height 4 years (cm)	1,288	103.51	5.10	1,307	102.84	5.05
Height 6 years (cm)	1,238	114.46	5.25	1,255	113.74	5.26
Height 7 years (cm)	1,249	120.35	5.65	1,303	119.65	5.50
Height 11 years (cm)	1,230	140.62	6.73	1,257	141.16	6.94
Height 15 years (cm)	1,135	162.04	8.86	1,156	158.65	6.22
Height 20 years (cm)	1,155	176.76	6.72	1,231	162.62	6.24
Weight 2 years (kg)	1,225	13.22	1.46	1,244	12.61	1.49
Weight 4 years (kg)	1,313	17.50	2.12	1,338	17.00	2.16
Weight 6 years (kg)	1,232	20.87	2.54	1,267	20.34	2.61
Weight 7 years (kg)	1,203	23.05	2.95	1,257	22.56	3.17
Weight 11 years (kg)	1,221	34.28	5.96	1,247	34.98	6.81
Weight 15 years (kg)	1,135	51.74	9.36	1,151	51.84	8.28
Weight 20 years (kg)	1,155	70.59	9.27	1,229	57.81	8.19
Birthweight (kg)	1,432	3.46	0.53	1,473	3.32	0.48

		Men		Women	
		N	%	n	%
Knee osteoarthritis at 53 years:		105	7.31	193	13.06
Sporting ability at 13 years:	Above average	235	18.98	220	17.31
	Average	793	64.05	902	70.97
	Below average	210	16.96	149	11.72
Father's occupational class in childhood:	Manual	605	43.71	600	42.43
	Non-manual	779	56.29	814	57.57

### Life course height and knee osteoarthritis

In sex-adjusted models, estimated associations between taller height and decreased odds of knee osteoarthritis at age 53 years were small at all ages. For example, the largest associations were an OR of knee osteoarthritis of 0.9 per 5 cm increase in height at age 4, (95% CI 0.7 to 1.1 (Model 1; Table II) and an OR of 0.9 per 5 cm increase in height, (95% CI 0.8 to 1.0) at age 6 (Table II). With adjustment for early life confounding factors (Model 2) and weight (Model 3), these estimates decreased further (Table II).

**Table II:** Associations between height (per 5 cm) at different ages throughout childhood, adolescence and young adulthood and odds ratios of knee osteoarthritis at age 53 years. Each set of models were run on the sample with valid data for knee osteoarthritis, height at the specific age and the confounders. Logistic regression Model 1: adjusted for sex; Model 2: further adjusted for birth weight, sporting ability and Father's occupational class in childhood; Model 3: further adjusted for weight at each age. Sex interactions: 2 years – *P* = 0.7; 4 years – *P* = 0.7; 6 years – *P* = 1.0; 7 years – *P* = 0.8; 11 years – *P* = 0.7; 15 years–0.8; 20 years – *P* = 0.09

Height (per 5 cm)	n	Model	Odds ratio	95% CI	
2 years	1,986	1	0.96	0.82	1.12
		2	0.98	0.84	1.14
		3	1.01	0.85	1.20
4 years	2,211	1	0.85	0.74	0.98
		2	0.87	0.75	1.01
		3	0.88	0.74	1.04
6 years	2,116	1	0.89	0.78	1.02
		2	0.91	0.79	1.05
		3	0.88	0.72	1.08
7 years	2,085	1	0.91	0.80	1.04
		2	0.94	0.83	1.08
		3	0.90	0.75	1.09
11 years	2,259	1	0.97	0.88	1.07
		2	0.99	0.89	1.10
		3	0.94	0.82	1.08
15 years	2,102	1	0.96	0.87	1.06
		2	0.98	0.89	1.09
		3	0.90	0.79	1.02
20 years	2,082	1	0.93	0.83	1.04
		2	0.95	0.85	1.07
		3	0.88	0.77	1.00

### Height growth and knee osteoarthritis

No associations were found between height gains during any of the four periods assessed and odds of knee osteoarthritis at 53 years (Table III). There was also no evidence of associations between height size, tempo or velocity (SITAR variables) and knee osteoarthritis at 53 years in models adjusted for sex and early life confounding factors (Models 1 & 2; Table IV). Increased SITAR height size and height tempo were marginally associated with lower odds of knee osteoarthritis at 53 years after additional adjustment SITAR weight size (Table IV).

**Table III:** Associations of conditional height gain (per standard deviation) during different periods of growth (early childhood: 2–4 years; late childhood: 4–7 years; childhood to adolescence: 7–15 years; adolescence to young adulthood: 15–20 years) with knee osteoarthritis at 53 years. Each set of models were run on the sample with valid data for knee osteoarthritis, conditional height gain during each life period, and the confounders. Logistic regression Model 1: adjusted for sex; Model 2: further adjusted for birth weight, sporting ability and Father's occupational class in childhood; Model 3: further adjusted for weight at each age. Sex interactions: 2–4 years – *P* = 0.2; 4–7 years – *P* = 0.6; 7–15 years – *P* = 0.3; 15–20 years – *P* = 0.1

Conditional change (per standard deviation)	n	Model	Odds ratio	95% CI	
2–4 years	1,876	1	0.91	0.78	1.07
		2	0.94	0.80	1.10
		3	0.91	0.77	1.08
4–7 years	1,689	1	0.94	0.80	1.10
		2	0.95	0.81	1.11
		3	0.95	0.80	1.13
7–15 years	1,710	1	1.09	0.93	1.30
		2	1.09	0.93	1.28
		3	0.99	0.83	1.18
15–20 years	1,611	1	1.05	0.89	1.23
		2	1.05	0.90	1.24
		3	0.99	0.84	1.17

**Table IV:** Associations between each parameter of the SITAR model of growth curve analysis (height size, tempo and velocity) and odds of knee osteoarthritis. Each set of models were run on the sample with valid data for knee osteoarthritis, each SITAR variable and the confounders. Logistic regression Model 1: adjusted for sex; Model 2: further adjusted for birth weight, sporting ability and Father's occupational class in childhood; Model 3: further adjusted for weight at each age. Sex interactions: size – *P* = 0.5; tempo – *P* = 0.8; velocity – *P* = 0.8

SITAR variable (*n* = 2,470)	Model	Odds ratio	95% CI	
Size (cm)	1	0.98	0.96	1.01
	2	0.99	0.97	1.01
	3	0.96	0.93	0.99
Tempo (%)	1	1.00	0.98	1.02
	2	0.99	0.98	1.01
	3	0.97	0.95	0.99
Velocity (%)	1	1.00	0.99	1.01
	2	1.00	0.99	1.02
	3	0.99	0.98	1.01

### Sensitivity analyses

Comparison of the characteristics of those individuals with complete data, vs those excluded are described in Tables S1.1 & S1.2. We found that higher proportions of those included were female (50.7% vs 49.3%; *P* < 0.001; Tables S1.1 & S1.2). No significant differences were observed in height between ages 2–15 years but at age 20, those included reported shorter heights (169.5 cm vs 171.0 cm) and lower weights (64.0 kg vs 65.5 kg) than those excluded (Table S1.1). When sex adjusted models were rerun on the maximum available samples including all available participants (Tables S2.1–S2.3), there were no substantive differences in findings. When we excluded those participants with potential secondary knee osteoarthritis from our analyses, there were no substantive differences in associations between height (Table S3.1), conditional height gain (Table S3.2), or SITAR variables (Table S3.3) and primary knee osteoarthritis at 53 years, compared with the main findings presented. Sex-stratified analyses confirmed that there were consistent patterns of association in men and women (Tables S4.1–4.3).

## Discussion

In this nationally representative British birth cohort study, associations between greater height at ages 4 and 6 years and marginally lower odds knee osteoarthritis at age 53 were observed in sex-adjusted models, but these were attenuated after adjustment for early life factors. No associations were observed between height changes during early childhood, late childhood, childhood to adolescence or adolescence to young adulthood or SITAR parameters and knee osteoarthritis.

A major strength of our study is the availability of multiple prospectively ascertained measurements of height throughout childhood and adolescence in the NSHD, together with the already derived SITAR variables and measures of knee osteoarthritis in a relatively large sample of people in midlife^9^. This provided a unique opportunity to investigate the associations between life course longitudinal growth and knee osteoarthritis at 53 years of age. Here we used two approaches to model growth and understand its relation to knee osteoarthritis in later life. Firstly, we used a conditional change approach to enable us to determine whether there are specific sensitive period/s of growth which may be associated with knee osteoarthritis. This can be interpreted as the change in height size above or below that expected given earlier height, and thus is useful in identifying accelerated or restricted growth^14^. We next chose the SITAR growth curve model since it was previously shown to effectively summarise pubertal growth based on three parameters of size, velocity and tempo^9^^,^^10^. A limitation of this approach is the use of multiple models which increases the chance of a type I error. Also, as in any longitudinal study, it is important to consider loss to follow-up over time and the impact of this on research findings. Despite losses to follow-up between birth and age 53 years, which may have introduced bias, comparisons with census data suggest that the respondent sample at age 53 were still representative of the general population born in the UK at a similar time in most respects^24^.

Our previous work explored associations between growth dynamics and osteoarthritis onset in a spontaneous murine model of osteoarthritis, the STR/Ort mouse^1^. We revealed accelerated long bone growth, aberrant expression of growth plate markers and enriched growth plate bridging, indicative of advanced and thus premature growth cessation, in these osteoarthritis-prone mice^1^. Together this suggested that these accelerated growth dynamics in young osteoarthritis-prone mice may underpin their osteoarthritis onset. However, whether these observations are unique to osteoarthritis in the STR/Ort mouse or are characteristic of human osteoarthritis in general had yet to be established. This study suggests that in the NSHD, associations between greater gains in height, indicative of accelerated growth, are not associated with increased odds of knee osteoarthritis. Rather, the modest associations found suggest the opposite. It is however important to note that this was examined in midlife when the cohort are still relatively young, and osteoarthritis prevalence (7.3% in men; 13.1% in women) is lower than that seen currently in primary care at this age. It would therefore be of interest to further examine these potential associations in older individuals.

Primary osteoarthritis is described as naturally occurring or ageing-related osteoarthritis, while secondary osteoarthritis is associated with other causes including trauma. Our previous findings in the STR/Ort mouse examined primary murine osteoarthritis^1^ and therefore to examine the influence of secondary knee osteoarthritis on the patterns of height growth in the NSHD, we ran a sensitivity analysis in which we excluded individuals who had reported consulting a Doctor about a knee injury. However, whilst we found no substantive differences in findings, this highlights the need to examine the risk of osteoarthritis in aged individuals where primary knee osteoarthritis is more prevalent.

Our study extends a previous study examining this British birth cohort in which prolonged exposure to high BMI through adulthood increased risk of development of knee osteoarthritis at age 53^3^. This is consistent with our sensitivity analyses in which adjustment for weight strengthened the associations between SITAR height size and odds of knee osteoarthritis. Wills *et al.*, also found that BMI increases from childhood to adolescence (7–15 years) were positively associated with knee osteoarthritis, however this was in women only^3^. In our analyses, we found no evidence of differences in association by sex. We did find that in our cohort with complete data, women had a higher prevalence of knee osteoarthritis, similar to that reported previously in the NSHD, and in primary care^3^^,^^15^. Wills *et al.*, concluded that the excessive weight during this period may result in altered mechanical loading to the knee joint. Similarly, it is likely that periods of accelerated growth will also impact on the biomechanics of the joint. The shape of the hip joint is largely determined in childhood, and previous studies have identified that in the NSHD, this is associated with (1) age of onset of walking in infancy^16^ (2) higher BMI at all ages and greater gains in BMI^17^ and (3) height, weight, BMI and BMD at ages 60–64 years^18^. Similarly, in the Avon Longitudinal Study of Parents and Children (ALSPAC) cohort, hip shape in perimenopausal women is associated with hip osteoarthritis susceptibility loci and may contribute to hip osteoarthritis later in life^19^. Recent evidence in the ALSPAC cohort has also identified pubertal timing, as reflected by height tempo, to be associated with hip shape^20^. Further, in the UK Biobank, early menarche is associated with higher risk for osteoarthritis^21^. However these associations were not observed in this study.

In conclusion, in this relatively large population-based cohort study, there was limited evidence to suggest that height in childhood is associated with odds of knee osteoarthritis at age 53 years. Further, there were no associations with height gain during specific periods of growth, or with the SITAR height growth variables. This work enhances our understanding of osteoarthritis predisposition and the contribution of life course height to this.

## Author contributions

All authors contributed to the conception and design of the study, or acquisition of data, or analysis and interpretation of data; drafting the article or revising it critically for important intellectual content and the final approval of the version to be submitted. KS (k.staines@brighton.ac.uk) takes responsibility for the integrity of the work as a whole, from inception to finished article.

## Conflict of interest

There are no conflicts of interest.

## Role of funding source

The authors would like to acknowledge the 10.13039/501100000265Medical Research Council for funding to KS (MR/R022240/1). The funding source was not involved in the study design, collection, analysis and interpretation of data; in the writing of the manuscript; or in the decision to submit the manuscript for publication.
