# Supplementary material for: Life course longitudinal growth and risk of knee osteoarthritis at age 53 years: evidence from the 1946 British birth cohort study
Source: Osteoarthritis Cartilage. 2021 Mar;29(3):335–40. doi: 10.1016/j.joca.2020.12.012 (PMC7955286; doi:10.1016/j.joca.2020.12.012)
Supplement: Multimedia component 1 [file mmc1.docx]

**S1: Cohort characteristics**

When compared with those excluded due to missing knee osteoarthritis and SITAR data, higher proportions of those included were female (50.7% vs 49.3%; p<0.001; Table S1.1). No differences were observed in height or weight between ages 2 – 15 years between those included vs those excluded. However, at age 20, those included reported slightly shorter heights than those excluded (171.0 cm vs 169.5 cm; Table S1.1). A higher proportion of those included had an average sporting ability (67.6% vs 63.4%) and were classed in a manual childhood social class (56.9% vs 43.07%), similar to that seen in those excluded (Table S1.1). Similar trends were found when comparing those excluded due to missing knee osteoarthritis, at least 1 height measure and all covariate data, with those included (Table S1.2).

|  | | **Included** | | | **Excluded** | | |
| --- | --- | --- | --- | --- | --- | --- | --- |
|  | | **N** | **Mean** | **SD** | **n** | **Mean** | **SD** |
| Height 2 years (cm) | | 2408 | 85.32 | 4.95 | 1602 | 85.32 | 5.21 |
| Height 4 years (cm) | | 2595 | 103.18 | 5.09 | 1689 | 103.04 | 5.25 |
| Height 6 years (cm) | | 2493 | 114.10 | 5.27 | 1568 | 114.03 | 5.46 |
| Height 7 years (cm) | | 2552 | 120.00 | 5.59 | 1588 | 119.90 | 5.75 |
| Height 11 years (cm) | | 2487 | 140.90 | 6.84 | 1508 | 140.57 | 7.06 |
| Height 15 years (cm) | | 2291 | 160.33 | 7.83 | 1348 | 160.31 | 8.22 |
| Height 20 years (cm) | | 2386 | 169.46 | 9.59 | 1255 | 170.98 | 9.80 |
| Weight 2 years (kg) | | 2469 | 12.91 | 1.52 | 1647 | 12.93 | 1.50 |
| Weight 4 years (kg) | | 2651 | 17.25 | 2.16 | 1738 | 17.21 | 2.20 |
| Weight 6 years (kg) | | 2499 | 20.60 | 2.59 | 1589 | 20.60 | 2.72 |
| Weight 7 years (kg) | | 2460 | 22.80 | 3.07 | 1540 | 22.82 | 3.24 |
| Weight 11 years (kg) | | 2468 | 34.64 | 6.42 | 1492 | 34.49 | 6.81 |
| Weight 15 years (kg) | | 2286 | 51.79 | 8.83 | 1331 | 51.85 | 9.84 |
| Weight 20 years (kg) | | 2384 | 64.00 | 10.82 | 1237 | 65.46 | 11.36 |
| Birthweight (kg) | | 2905 | 3.39 | 0.51 | 2422 | 3.36 | 0.58 |
|  | | **n** | **%** |  | **n** | **%** |  |
| Knee osteoarthritis | | 298 | 10.22 |  | 4 | 9.52 |  |
| Sporting ability: | Above average | 455 | 18.13 |  | 279 | 18.54 |  |
|  | Average | 1695 | 67.56 |  | 954 | 63.39 |  |
|  | Below average | 359 | 14.31 |  | 272 | 18.07 |  |
| Childhood Social class: | Non-manual | 1205 | 43.07 |  | 721 | 38.45 |  |
|  | Manual | 1593 | 56.93 |  | 1154 | 61.55 |  |

***Table S1.1*** *Comparison of the* *characteristics of individuals from the MRC National Survey of Health and Development with complete data on the SITAR parameters of height and the outcome, knee osteoarthritis, vs those excluded.*

|  | | **Included** | | | **Excluded** | | |
| --- | --- | --- | --- | --- | --- | --- | --- |
|  | | **n** | **Mean** | **SD** | **n** | **Mean** | **SD** |
| Height 2 years (cm) | | 2065 | 85.31 | 4.93 | 1945 | 85.33 | 5.19 |
| Height 4 years (cm) | | 2257 | 103.11 | 5.06 | 2027 | 103.14 | 5.26 |
| Height 6 years (cm) | | 2211 | 114.09 | 5.24 | 1850 | 114.04 | 5.46 |
| Height 7 years (cm) | | 2301 | 120.04 | 5.55 | 1839 | 119.86 | 5.77 |
| Height 11 years (cm) | | 2290 | 140.89 | 6.84 | 1705 | 140.62 | 7.04 |
| Height 15 years (cm) | | 2132 | 160.30 | 7.87 | 1507 | 160.35 | 8.12 |
| Height 20 years (cm) | | 2121 | 169.48 | 9.58 | 1520 | 170.70 | 9.79 |
| Weight 2 years (kg) | | 2117 | 12.89 | 1.49 | 1999 | 12.96 | 1.52 |
| Weight 4 years (kg) | | 2310 | 17.21 | 2.14 | 2079 | 17.26 | 2.20 |
| Weight 6 years (kg) | | 2219 | 20.58 | 2.57 | 1869 | 20.62 | 2.72 |
| Weight 7 years (kg) | | 2216 | 22.77 | 3.06 | 1784 | 22.86 | 3.24 |
| Weight 11 years (kg) | | 2270 | 34.57 | 6.34 | 1690 | 34.59 | 6.86 |
| Weight 15 years (kg) | | 2128 | 51.76 | 8.91 | 1489 | 51.88 | 9.63 |
| Weight 20 years (kg) | | 2119 | 63.98 | 10.84 | 1502 | 65.23 | 11.25 |
| Birthweight (kg) | | 2470 | 3.40 | 0.51 | 2857 | 3.36 | 0.57 |
|  | | **n** | **%** |  | **n** | **%** |  |
| Knee osteoarthritis | | 247 | 10.00 |  | 55 | 11.30 |  |
| Sporting ability: | Above average | 448 | 18.14 |  | 286 | 18.52 |  |
|  | Average | 1669 | 67.57 |  | 980 | 63.47 |  |
|  | Below average | 353 | 14.29 |  | 278 | 18.01 |  |
| Childhood social class: | Non-manual | 1034 | 41.86 |  | 892 | 40.49 |  |
|  | Manual | 1436 | 58.14 |  | 1311 | 59.51 |  |

***Table S1.2*** *Comparison of the* *characteristics of individuals from the MRC National Survey of Health and Development with complete data on the outcome, knee osteoarthritis, at least one height variables and all covariates, vs those excluded.*

**S2: Maximal data sets**

Additional sex-adjusted analyses were run in which the associations were examined including all available participants rather than being restricted to the sample with valid data on all covariates. Tables S2.1, S2.2 & S2.3 show that the results were similar to those presented in the main manuscript, both in terms of the patterns of associations and the sex interactions.

| **Height (per 5cm)** | **n** | **Odds ratio** | **95% CI** | |
| --- | --- | --- | --- | --- |
| 2 years | 2408 | 0.95 | 0.83 | 1.09 |
| 4 years | 2597 | 0.87 | 0.77 | 0.99 |
| 6 years | 2493 | 0.90 | 0.79 | 1.01 |
| 7 years | 2487 | 0.97 | 0.89 | 1.07 |
| 11 years | 2487 | 0.99 | 0.98 | 1.01 |
| 15 years | 2291 | 0.99 | 0.90 | 1.08 |
| 20 years | 2388 | 0.94 | 0.85 | 1.05 |

***Table S2.1:*** *Associations between height (per 5cm) at different ages throughout childhood, adolescence and young adulthood and odds ratios of knee osteoarthritis at age 53 years, using the maximal sample (all available participants). Logistic regression adjusted for sex. Sex interactions: 2 years – p=0.2; 4 years – p=0.8; 6 years – p=1.0; 7 years – p=0.5; 11 years – p=0.5; 15 years – 0.6; 20 years – p=0.1.*

| **Conditional change (per standard deviation)** | **n** | **Odds ratio** | **95% CI** | |
| --- | --- | --- | --- | --- |
| 2 - 4 years | 2266 | 0.89 | 0.78 | 1.02 |
| 4 - 7 years | 2062 | 0.96 | 0.84 | 1.11 |
| 7 - 15 years | 1980 | 1.14 | 0.99 | 1.32 |
| 15 - 20 years | 1857 | 1.04 | 0.89 | 1.20 |

***Table S2.2:*** *Associations of conditional height gain (per standard deviation) during different periods of growth (early childhood: 2–4 years; late childhood: 4-7 years; childhood to adolescence: 7–15 years; adolescence to young adulthood: 15–20 years) with knee osteoarthritis at 53 years, using the maximal sample (all available participants).* *Logistic regression adjusted for sex. Sex interactions: 2-4 years – p=0.7; 4-7 years – p=0.8; 7-15 years – p=0.8; 15-20 years – p=0.05.*

| **SITAR variable**  **n=2915** | **Odds ratio** | **95% CI** | |
| --- | --- | --- | --- |
| Size (cm) | 0.99 | 0.97 | 1.01 |
| Tempo (%) | 0.99 | 0.98 | 1.01 |
| Velocity (%) | 1.01 | 0.99 | 1.02 |

***Table S2.3:*** *Associations between each parameter of the SITAR model of growth curve analysis (height size, tempo and velocity) and odds of knee osteoarthritis, using the maximal sample (all available participants). Logistic regression adjusted for sex. Sex interactions: size – p=0.3; tempo – p=0.7; velocity – p=0.6.*

**S3: Influence of secondary knee osteoarthritis**

To examine the influence of secondary knee osteoarthritis on the patterns of height growth, we repeated the analysis after excluding individuals with valid data for the SITAR parameters of height and knee osteoarthritis, who had reported consulting a Doctor about a knee injury, if that knee injury was on the same limb as that diagnosed with osteoarthritis. Tables S3.1, S3.2 & S3.3 show that the results were similar to those presented in the main manuscript.

| **Height (per 5cm)** | **n** | **Model** | **Odds ratio** | **95% CI** | |
| --- | --- | --- | --- | --- | --- |
| 2 years | 1914 | 1 | 0.94 | 0.78 | 1.14 |
|  |  | 2 | 0.97 | 0.80 | 1.18 |
|  |  | 3 | 0.98 | 0.80 | 1.21 |
| 4 years | 2134 | 1 | 0.81 | 0.69 | 0.96 |
|  |  | 2 | 0.84 | 0.71 | 1.00 |
|  |  | 3 | 0.83 | 0.67 | 1.02 |
| 6 years | 2045 | 1 | 0.80 | 0.70 | 0.95 |
|  |  | 2 | 0.83 | 0.70 | 0.98 |
|  |  | 3 | 0.77 | 0.61 | 0.99 |
| 7 years | 2009 | 1 | 0.95 | 0.84  83 | 1.09 |
|  |  | 2 | 0.98 | 0.86 | 1.12 |
|  |  | 3 | 0.98 | 0.82 | 1.18 |
| 11 years | 2177 | 1 | 0.99 | 0.96 | 1.01 |
|  |  | 2 | 0.99 | 0.97  9  97 | 1.02 |
|  |  | 3 | 0.98 | 0.94 | 1.01 |
| 15 years | 2028 | 1 | 0.95 | 0.84 | 1.07 |
|  |  | 2 | 0.98 | 0.87 | 1.11 |
|  |  | 3 | 0.87 | 0.75 | 1.01 |
| 20 years | 2007 | 1 | 0.92 | 0. 80 | 1.06 |
|  |  | 2 | 0.92 | 0.80 | 1.06 |
|  |  | 3 | 0.86 | 0.73 | 1.00 |

***Table S3.1:*** *Associations between height (per cm) at different ages throughout childhood, adolescence and young adulthood and odds ratios of primary knee osteoarthritis at age 53 years. Each set of models were run on the sample with valid data for primary knee osteoarthritis, height at the specific age and the confounders. Logistic regression Model 1: adjusted for sex; Model 2: further adjusted for birth weight, sporting ability and father’s occupational class in childhood; Model 3: further adjusted for weight at each age. There was little evidence of sex interaction in any of these models: P>0.09. Sex interactions: 2 years – p=0.5; 4 years – p=0.6; 6 years – p=0.9; 7 years – p=0.8; 11 years – p=0.8; 15 years – 0.9; 20 years – p=0.4.*

| **Conditional change (per standard deviation)** | **n** | **Model** | **Odds ratio** | **95% CI** | |
| --- | --- | --- | --- | --- | --- |
| 2 - 4 years | 1808 | 1 | 0.89 | 0.74 | 1.08 |
|  |  | 2 | 0.94 | 0.77 | 1.14 |
|  |  | 3 | 0.91 | 0.74 | 1.13 |
| 4 - 7 years | 1628 | 1 | 0.87 | 0.71 | 1.06 |
|  |  | 2 | 0.89 | 0.73 | 1.08 |
|  |  | 3 | 0.87 | 0.71 | 1.08 |
| 7 - 15 years | 1648 | 1 | 1.12 | 0.92 | 1.37 |
|  |  | 2 | 1.12 | 0.92 | 1.36 |
|  |  | 3 | 1.01 | 0.82 | 1.25 |
| 15 - 20 years | 1549 | 1 | 1.01 | 0.83 | 1.22 |
|  |  | 2 | 1.02 | 0.84 | 1.24 |
|  |  | 3 | 1.00 | 0.81 | 1.23 |

***Table S3.2:*** *Associations of conditional height gain (per standard deviation) during different periods of growth (early childhood: 2–4 years; late childhood: 4-7 years; childhood to adolescence: 7–15 years; adolescence to young adulthood: 15–20 years) with primary knee osteoarthritis at 53 years. Each set of models were run on the sample with valid data for primary knee osteoarthritis, conditional height gain during each life period, and the confounders.*  *Logistic regression Model 1: adjusted for sex; Model 2: further adjusted for birth weight, sporting ability and father’s occupational class in childhood; Model 3: further adjusted for weight at each age. Sex interactions: 2-4 years – p=0.3; 4-7 years – p=0.6; 7-15 years – p=0.7; 15-20 years – p=0.2.*

| **SITAR variable (n=2385)** | **Model** | **Odds ratio** | **95% CI** | |
| --- | --- | --- | --- | --- |
| Size (cm) | 1 | 0.97 | 0.95 | 1.00 |
|  | 2 | 0.98 | 0.95 | 1.01 |
|  | 3 | 0.95 | 0.92 | 0.98 |
| Tempo (%) | 1 | 0.99 | 0.97 | 1.02 |
|  | 2 | 0.99 | 0.97 | 1.01 |
|  | 3 | 0.97 | 0.95 | 1.00 |
| Velocity (%) | 1 | 1.00 | 0.99 | 1.02 |
|  | 2 | 1.00 | 0.99 | 1.02 |
|  | 3 | 0.99 | 0.98 | 1.01 |

***Table S3.3:*** *Associations between each parameter of the SITAR model of growth curve analysis (height size, tempo and velocity) and odds of primary knee osteoarthritis. Each set of models were run on the sample with valid data for knee osteoarthritis, each SITAR variable and the confounders. Logistic regression Model 1: adjusted for sex; Model 2: further adjusted for birth weight, sporting ability and father’s occupational class in childhood; Model 3: further adjusted for weight at each age. Sex interactions: size – p=0.7; tempo – p=0.7; velocity – p=0.5.*

**S4. Sex stratified analyses**

Sex stratified analyses revealed similar results to those presented in the main manuscript.

| **Height (per 5cm)** | **Sex** | **n** | **Odds ratio** | **95% CI** | |
| --- | --- | --- | --- | --- | --- |
| 2 years | M | 1004 | 0.92 | 0.73 | 1.16 |
|  | F | 982 | 0.99 | 0.80 | 1.22 |
| 4 years | M | 1102 | 0.88 | 0.70 | 1.11 |
|  | F | 1109 | 0.84 | 0.71 | 1.00 |
| 6 years | M | 1045 | 0.89 | 0.71 | 1.13 |
|  | F | 1071 | 0.89 | 0.75 | 1.06 |
| 7 years | M | 1024 | 0.97 | 0.81 | 1.16 |
|  | F | 1061 | 0.99 | 0.87 | 1.13 |
| 11 years | M | 1113 | 0.99 | 0.96 | 1.02 |
|  | F | 1146 | 1.00 | 0.97 | 1.02 |
| 15 years | M | 1039 | 0.95 | 0.83 | 1.08 |
|  | F | 1063 | 0.98 | 0.85 | 1.13 |
| 20 years | M | 1004 | 0.83 | 0.69 | 0.98 |
|  | F | 1078 | 1.00 | 0.87 | 1.16 |

***Table S4.1:*** *Associations between height (per 5cm) at different ages throughout childhood, adolescence and young adulthood and odds ratios of knee osteoarthritis at age 53 years. Logistic regression.*

| **Conditional change (per standard deviation)** | **Sex** | **n** | **Odds ratio** | **95% CI** | |
| --- | --- | --- | --- | --- | --- |
| 2 - 4 years | M | 958 | 1.04 | 0.80 | 1.36 |
|  | F | 918 | 0.85 | 0.70 | 1.03 |
| 4 - 7 years | M | 856 | 0.88 | 0.66 | 1.17 |
|  | F | 833 | 0.96 | 0.79 | 1.17 |
| 7 - 15 years | M | 856 | 0.97 | 0.74 | 1.27 |
|  | F | 854 | 1.16 | 0.97 | 1.41 |
| 15 - 20 years | M | 779 | 0.88 | 0.67 | 1.16 |
|  | F | 832 | 1.14 | 0.94 | 1.38 |

***Table S4.2:*** *Associations of conditional height gain (per standard deviation) during different periods of growth (early childhood: 2–4 years; late childhood: 4-7 years; childhood to adolescence: 7–15 years; adolescence to young adulthood: 15–20 years) with knee osteoarthritis at 53 years.* *Logistic regression.*

| **SITAR variable** | **Sex** | **n** | **Odds ratio** | **95% CI** | |
| --- | --- | --- | --- | --- | --- |
| Size (cm) | M | 1217 | 0.97 | 0.94 | 1.01 |
|  | F | 1253 | 0.99 | 0.96 | 1.02 |
| Tempo (%) | M | 1217 | 0.99 | 0.96 | 1.03 |
|  | F | 1253 | 1.00 | 0.98 | 1.02 |
| Velocity (%) | M | 1217 | 1.00 | 0.98 | 1.02 |
|  | F | 1253 | 1.00 | 0.99 | 1.02 |

***Table S4.3:*** *Associations between each parameter of the SITAR model of growth curve analysis (height size, tempo and velocity) and odds of knee osteoarthritis. Logistic regression.*
